# Augmenting the antinociceptive effects of nicotinic acetylcholine receptor activity through *lynx1* modulation

**DOI:** 10.1371/journal.pone.0199643

**Published:** 2018-07-03

**Authors:** Neel I. Nissen, Kristin R. Anderson, Huaixing Wang, Hui Sun Lee, Carly Garrison, Samantha A. Eichelberger, Kasarah Ackerman, Wonpil Im, Julie M. Miwa

**Affiliations:** 1 Department of Biological Science, Lehigh University, Bethlehem, PA, United States of America; 2 Department of Health Science and Technology, Aalborg University, Aalborg, Denmark; Weizmann Institute of Science, ISRAEL

## Abstract

Neuronal nicotinic acetylcholine receptors (nAChRs) of the cholinergic system have been linked to antinociception, and therefore could be an alternative target for pain alleviation. nAChR activity has been shown to be regulated by the nicotinic modulator, lynx1, which forms stable complexes with nAChRs and has a negative allosteric action on their function. The objective in this study was to investigate the contribution of lynx1 to nicotine-mediated antinociception. *Lynx1* contribution was investigated by mRNA expression analysis and electrophysiological responses to nicotine in the dorsal raphe nucleus (DRN), a part of the pain signaling pathway. *In vivo* antinociception was investigated in a test of nociception, the hot-plate analgesia assay with behavioral pharmacology. *Lynx1*/α4β2 nAChR interactions were investigated using molecular dynamics computational modeling. Nicotine evoked responses in serotonergic and GABAergic neurons in the DRN are augmented in slices lacking *lynx1* (*lynx1*KO). The antinociceptive effect of nicotine and epibatidine is enhanced in *lynx1*KO mice and blocked by mecamylamine and DHβE. Computer simulations predict preferential binding affinity of *lynx1* to the α:α interface that exists in the stoichiometry of the low sensitivity (α4)_3_(β2)_2_ nAChRs. Taken together, these data point to a role of *lynx1* in mediating pain signaling in the DRN through preferential affinity to the low sensitivity α4β2 nAChRs. This study suggests that *lynx1* is a possible alternative avenue for nociceptive modulation outside of opioid-based strategies.

## Introduction

Pain is amongst the most common reasons for seeking medical treatment, however approximately 80 percent of pain sufferers worldwide do not have sufficient access to proper care [1–2]. The most widely used therapies for acute nociceptive pain involve agonists of the opioid receptors. Such agents, however, carry risk for major off-target effects [3], along with the high propensity for overdose, abuse and the development of tolerance [4], which is a rapidly growing medical and societal concern in the US [5]. Analgesia can be achieved by influencing pathways other than the opioidergic pathways [6] and one promising alternate avenue outside of opioid agents is to exploit the antinociceptive effects of the cholinergic system, more specifically neuronal nicotinic acetylcholine receptors (nAChRs) of this neurotransmitter system.

Stimulation of nAChRs have been shown to produce antinociception by several pharmacological studies [7–12]. nAChRs are activated by the endogenous neurotransmitter acetylcholine and the exogenous drug nicotine [13]. Neuronal nAChRs exist in combinations of either heteropentameric or homopentameric complexes of α2-10and β2–4 nAChR subunits and the majority of nAChRs in the brain consist of α4β2 heteropentamers or α7 homopentamers [14–17].The subunit composition and stoichiometry of nAChRs affect the response profile of the receptor [18] and studies on acute pain have shown that specifically, α4β2 [7–12] α5 [19], α6*-containing [20], α7 [21–23] and α9*-containing [24] nAChR subtypes are important components in the nAChR-mediated antinociception pathway [25].

Pain signaling emanates from the periphery and involves the spinal cord [26], periaqueductal gray and dorsal raphe nucleus (DRN) etc. [27]. nAChRs can directly modulate serotonergic neurons in the DRN to influence nociception, resulting in antinociceptive activity [28–30]. Extracellular nicotine has been shown to elicit an increase in the firing rate of most DRN neurons, thus enhancing serotonin release causing antinociception [29–30]. Another region of interest in the pain signaling is the dorsal horn of the spinal cord, which also expresses nAChRs. Spinal nAChRs have been shown to have both nociceptive and antinociceptive roles [31]. Endogenous acetylcholine in the spinal cord tonically drives baseline signaling, which can alter the thresholds for pain [32]. The periaqueductal gray is a relay station between brain regions such as the hypothalamus and the spinal cord that contains α7 nAChRs, and when locally activated, these α7 receptors can cause antinociception [33]. Peripheral nAChRs in the dorsal root ganglion neurons also carries nociceptive and antinociceptive sensory signaling [34–35]. The use of α4β2 and α7 nAChR agonists in this area induce antinociception [36–38]. Thus, modulation of nAChRs expressed in these pathways can contribute to antinociception.

Protein modulators of nAChRs represent an avenue of investigation into the role of nAChRs in antinociception. Lynx genes belong to the Ly-6/uPAR superfamily [39] which are evolutionarily related to genes coding for snake venom toxins. Like snake venom toxins and other prototoxins [40–43], the *lynx1* protein binds to nAChRs and modulates their function [44]. Both snake venom toxins and *lynx1* proteins have a three-looped binding fold which is an efficient receptor binding scaffold [45–46]. *Lynx1* has been shown to form stable complexes with both α4β2 and α7 nAChR subunits on CNS neurons [44] and through its binding to nAChRs, as early as in the endoplasmatic reticulum [47], it can have overall complex effects on nicotinic receptor function; *lynx1* binding results in reduced agonist sensitivity, increased desensitization rate, and slower recovery from desensitization of nAChRs [48], and increase in the mean closed time measured at the single-channel level [49]. The biophysical mechanism of action of *lynx1* on nAChR function, and the widespread expression of nAChRs, suggest a complex, multimodal role of lynx1 on nAChR-dependent processes. The functional *in vivo* consequences have been tested in *lynx1* knockout (KO) mice, and thus far, increased associate learning, improved memory [48] and extended critical period of plasticity in the visual system [50], have been reported. Although this establishes the functional significance of these modulatory proteins, other nicotinic-dependent processes such as antinociception have yet to be investigated. With the detection of *lynx1* expression in brain regions linked to nociception processing reported herein, we sought to assess nAChR-mediated antinociception.

## Materials and methods

### Model organism

Genetically modified female and male mice were used as the model organism for this study. Both wild type (wt) (C57BL/6J), *lynx1*KO and β2-GFP knockin mice were used. The mice tested were between the ages of 3–7 months (20-50g) for the nicotine-, epibatidine- and dihydro-β-erythroidine hydrobromide (DHβE) assays and 6–8 months (30-50g) for the mecamylamine (mec)-, locomotion- and temperature assays. Naïve mice were used for the nicotine-, epibatidine- and DHβE assays, and reused for the mec-, locomotion- and temperature assays with sufficient time in between assays (minimum 1 week). Adult naïve mice were used for PCR, immunohistochemical staining and western blot analysis. For the electrophysiological experiment 16–18 days old naïve mice were used. The *lynx1*KO and β2-GFP mice were transferred from the California Institute of Technology in Pasadena, CA, USA or bred at Lehigh University, PA, USA. *Lynx1*KO mice were generated using 129 ES cells [48] and backcrossed over 12 generations to the C57BL/6J background. β2-GFP knock-in mice were generated in the Lester laboratory, California Institute of Technology in Pasadena, CA, USA, and transferred to the Lehigh Central Animal Facility. Mice were kept on a 12:12 light-dark cycle and food and water were provided ad libitum. The mice were kept with up to 4 other mice and were ear punched for identification purposes. All procedures and protocols were approved and in compliance with Lehigh IACUC guidelines on the humane care and use of animals (IACUC protocol #147). All efforts were made to minimize animal suffering. Animal studies are reported in compliance with ARRIVE guidelines.

### Genotyping

All mice were genotyped using a polymerase chain reaction assay. A mouse tail biopsy was obtained before and after experimentation for both pre- and post-hoc genotyping. The DNA was lysed overnight with Proteinase K and extracted using isopropanol or Qiagen© DNA Miniprep Kit (Qiagen, Hilden, Germany). The DNA was amplified through a standard polymerase chain reaction protocol. Each tissue sample was genotyped 2–3 times before a confirmation of the genotype was assigned.

### Quantitative PCR and RT-PCR

Tissue from the DRN was dissected and RNA was extracted using the RNeasy Mini kit (Qiagen, Hilden, Germany). Purified RNA was synthesized into cDNA using the qScript cDNA SuperMix (Quanta Bioscience, Beverly, MA, USA). Quantitative PCR was performed in triplicate using *lynx1* TaqMan gene expression assay (ThermoFisher Scientific, Waltham, MA, USA) with GAPDH as reference gene. The expression of target genes was normalized to the expression of the reference gene [51]. PCR products were run on a 4% agarose gel. Furthermore, RT-PCR was also carried out using tryptophan hydroxylase 2 primers (FW: 5’CTGAAAGAGCGATCTGGCTTC3’, Rev: 5’ATCTGGTTCCGGGGTGTAGA3’) (Integrated DNA technologies, USA) to confirm localization of RNA to the DRN. PCR products were run on a 2% agarose gel.

### *Lynx1* immunohistochemical staining

Adult β2-GFP knockin mice were anesthetized and perfused with saline followed by 4% paraformaldehyde/PBS (pH 7.4) followed by rapid decapitation. Perfused brains were dissected, and post-fixed in 4% paraformaldehyde/PBS for 3 hours followed by 30% sucrose/PBS. Dissected brains were sectioned at 50 μm on a freezing microtome. Experiments were done by incubating free-floating sections. Sections were stained with custom made anti-*lynx1* monoclonal antibodies (1:1000, 1.7 ng/μl)[44]) o/n, and incubated with Alexa Fluor 568 conjugated anti-mouse secondary antibodies at 1:1000 dilution for 1 hour at RT (ThermoFisher Scientific, Waltham, MA, USA). Serotonergic neurons were identified using an anti-TPH2 (Tryptophan hydroxylase 2) antibody (1:500, ThermoFisher Scientific, Waltham, MA, USA) o/n, and incubated with Cy-2 conjugated donkey anti-rabbit secondary antibodies at 1:2000 dilution (Jackson Immunochemicals, West Grove, PA, USA) at RT for 1 hour. Sections were also incubated with rabbit polyclonal antibodies against *lynx1* ([39]. These antibodies were custom generated against a synthetic *lynx1* peptide (TTRTYFTPYRMKVRKS) and previously tested by immunofluorescence and immunocytochemistry staining at 1:2000 dilution [39]. To confirm the specificity of staining of this antisera, affinity-purified anti-*lynx1* antibodies were tested by Western blot of mouse brain extracts to confirm ~9–12 kD band size, using antibodies purified on a nickel column to purify a bacterially produced *lynx1*-his protein, and subsequently tested on Western blots from *lynx1*KO mouse brain extracts. The staining in the *lynx1* +/+ mouse brains demonstrated band at the expected band size, and no band was present in the *lynx1*KO -/- brain samples [48]. DRN sections were incubated with anti-*lynx1* antisera at 1:1000 dilution for 1 hour at RT. After washing, sections were incubated with Cy-2-conjugated donkey anti rabbit at 1:200 (Jackson Immunoresearch, West Grove, PA, USA) for 1 hour at RT. Sections were mounted on microscope slides and imaged using Nikon elements software on Nikon E1000 confocal microscope.

### Dual labeling immunofluorescence

Adult C57bl6 mice were perfused as described above. Dissected brains were sectioned at 50μm on a freezing microtome. Serotonergic neurons were identified using an anti-TPH2 (Tryptophan hydroxylase 2) antibody (1:500, ThermoFisher Scientific, Waltham, MA, USA) o/n, and incubated with Cy-2 conjugated donkey anti-rabbit secondary antibodies at 1:2000 dilution (Jackson Immunochemicals, West Grove, PA, USA) at RT for 1 hour. Sections were co-stained with custom made anti-*lynx1* monoclonal antibodies raised against mouse lynx1 protein purified from bacteria and refolded (Green Mountain Laboratories, mouse, 1:1000, 1.7 ng·μl^-1^) [44] o/n, and incubated with Alexa Fluor 568 conjugated anti-mouse secondary antibodies at 1:1000 dilution for 1 hour at RT (ThermoFisher Scientific, Waltham, MA, USA). Sections were mounted and imaged using Nikon elements software on Nikon E1000 confocal microscope at 10, 20, 40 and 100x magnification. Sections were also stained singly with either anti-TPH2 or anti-*lynx1* antibodies to establish staining pattern and control for bleed through staining. To further confirm the specificity of *lynx1* staining, we stained side by side DRN sections with anti-*lynx1* polyclonal and anti-TPH2 antibodies.

### Co-immunoprecipitation (Co-IP)

β2-GFP knockin dorsal brain regions containing the DRN were dissected and immediately homogenized in the bullet blender tissue homogenizer (NextAdvance, Averill Park, NY, USA), with Co-IP buffer (50 mM Tris, 150 mM NaCl, 0.75% Triton-X 100, Pierce protease inhibitor cocktail). Dynabeads A (ThermoFisher Scientific, Waltham, MA, USA) were pre-incubated with 10μg rabbit anti-GFP primary antibodies (ThermoFisher Scientific,Waltham, MA, USA) and thoroughly washed. Brain homogenates at a concentration of 13 mg·ml^-1^ were incubated with the dynabeads-antibody complex for 3 days rotating at RT. After washing, GFP fusion protein complexes including interacting proteins were eluted and immediately prepared for western blot analysis.

### Electrophysiology

Animals, aged 16–18 days, were split into 2 groups according to their genotype. The mice were deeply anaesthetized with isoflurane. Frontal midbrain slices at 300 μm were made with a Vibratome (Vibratome Co., St Louis, MO, USA), and the slices were incubated in an oxygenated sucrose solution at 35°C for 1 hour. The recordings were perfused with Ringer solution containing the following ingredients (in mM): NaCl 128, KCl 2.5, NaH2PO4 1.25, CaCl2 2, MgSO4 1, NaHCO3 26, and dextrose 10, pH 7.4. Whole-cell patch clamp recordings were conducted in DRN slices visualized through an upright microscope (Scientifica, UK) equipped with infrared differential interference contrast optics (IR-DIC). Resistance of the recording pipette (1.2 mm borosilicate glass) was 7–9 MΩ. Tips of the recording pipettes were filled with a potassium gluconate-based intracellular solution: potassium gluconate 120, KCl 6, ATP-Mg 4, Na2GTP 0.3, EGTA 0.1, and Hepes 10, pH 7.3, 310 mosmol·L^-1^. Cell type identification using the action potential (AP) shoulder was performed under current clamp recording conditions to discriminate serotonergic neurons (with shoulder) and GABAergic neurons (without a shoulder). The cell was then switched into voltage clamp mode and held at -70 mV for the assessment of nicotine-evoked peak responses. Nicotine was dissolved in ACSF for fast-application to the neuronal cell body using a Picospritzer (20 p.s.i., 200 ms) to pressure-eject the nicotine solution (Parker Hannifin corporation). The peak value of response was measured and statistically analyzed among different groups.

### Western blot analyses

Samples were denatured in 1x sample buffer (ThermoFisher Scientific, Waltham, MA, USA) at 95°C and run on a 15% SDS-PAGE gel. Gels were transferred onto activated PVDF membrane via semi-dry transfer. The membrane was blocked with 5% milk/0.05% Tween-PBS for 1 hour at 4°C followed by an overnight incubation at 4°C in T-15 anti-lynx1 (1:1000, Santa Cruz Biotechnology, Dallas, TX, USA). After thorough washing, the membrane was incubated with conjugated donkey anti-goat (Abcam, Cambridge, MA, USA) at 1:10,000 for 1 hour at 4°C. Membranes were incubated in ECL (ThermoFisher Scientific,Waltham, MA, USA) and exposed to film. Actin controls were run using mouse anti-actin (Abcam, Cambridge, MA, USA) at 1:1000 dilution, and goat anti-mouse (Life Technologies, Carlsbad, CA, USA) at 1:40,000 dilution.

### Drugs

Nicotine hydrogen tartrate salt (free base nicotine concentrations of 0.25^−1^.5 mg·kg^-1^) (Sigma-Aldrich, St. Louis, MO, USA), mec (2.5 mg·kg^-1^) (Abcam, Cambridge, MA, USA), DHβE (3.0 mg·kg^-1^) (Tocris Bioscience, Bristol, UK), epibatidine (5 μg·kg^-1^) (Tocris Bioscience, Bristol, UK), and Ibuprofen sodium (20 mg·kg^-1^) (Sigma-Aldrich, St. Louis, MO, USA) were dissolved in 0.9% saline. The volume of liquid injected into the mice was calculated based on animal weight of ~10 ml·kg^-1^ [52]. The control animals were injected with 0.9% saline.

### Hot-plate assay

The hot-plate test was used to measure response latencies according to a method previously described [53]. The hot-plate (Columbus Instruments, Columbus, OH, USA) was set at a temperature of 55°C, and the latency to lick the hind-paw or jump off the surface was evaluated as an index of nociception. Immediate signs of temperature sensation such as fluttering of the feet or licking of the front paws were also noted, however the mice were not removed from the hot-plate until jumping or hind-paw licking. A cutoff time of 60 seconds was determined [11]. Mice were examined and no tissue damage post experiment was observed. If a mouse reached 60 seconds on the hot-plate without demonstrating one of our two proposed nociceptive indicators, the mouse was removed from the hot-plate and a time of 60 seconds was recorded. Mice were injected with either saline (n = 8, 8; wt, *lynx1*KO, respectively), free base nicotine concentrations of 0.25 mg·kg^-1^ (n = 8, 8) 0.5 mg·kg^-1^ (n = 8, 9), 1.0mg·kg^-1^ (n = 8, 14) and 1.5mg·kg^-1^ (n = 8, 8) epibatidine, 5 μg·kg^-1^ (n = 16, 13), or ibuprofen 20 mg·kg^-1^ [54] (n = 10, 12)15 minutes prior to testing on the hot-plate. For the nAChR blocker mecamylamine (mec), mice were given an injection of 2.5 mg·kg^-1^ (n = 16, 13) 15 minutes prior to hot-plate testing. Ten minutes after mec injection the mice were injected with nicotine (1 mg·kg^-1^). The β2*-specific nAChRs inhibitor dihydro-β-erythroidine hydrobromide (DHβE) were injected IP at a dose of 3.0 mg·kg^-1^ (n = 6, 6) 25 minutes prior to hot-plate testing. Ten minutes after DHβE injection mice were injected with nicotine (0.5 mg·kg^-1^). Animals were split into groups according to their genotype and randomized with respect to males and females.

Locomotion Assay: Wt and *lynx1*KO mice were injected with the same free base nicotine concentrations as in the hot-plate test (saline n = 5,6; wt, *lynx1*KO, respectively, 0.5 mg·kg^-1^ n = 7, 8, 1.0 mg·kg^-1^ n = 6, 6 and 1.5 mg·kg^-1^ n = 6, 6). Motor activity was assessed by videotaping and measuring the amount of leg movements in seconds in the time period 15–20 minutes post injections. In addition, leg movements in the time period 5–10, 10–15, and 15–20 minutes post injection for saline (n = 5 wt, 6 L1KO) and 0.5 mg·kg^-1^ (n = 3 wt, 3 L1KO) to confirm that no difference in locomotor activity between genotypes was observed at any relevant time period.

### Body temperature assay

Wt and *lynx1*KO mice were injected with either saline (n = 8, 11; wt, *lynx1*KO, respectively) or free base nicotine (1.0 mg·kg^-1^ n = 8, 11, and 2.5 mg·kg^-1^ n = 7, 7). 15 minutes before and after injections temperature was measured in degrees Celsius (°C) using a 20 mm rectal probe for mice (BrainTree Scientific Inc., Braintree, MA, USA).

### Computer modeling of *lynx1*-AChR complexes

A low-resolution structure model of α4:α4/*lynx1* complex was first generated using a template-based approach. The structure of α4 subunit and that of *lynx1* were taken from the Protein Data Bank (PDB) entries 5kxi [55] and 2l03 [45], respectively. Each target structure (α4 subunit or *lynx1*) was searched against all known proteins in the PDB to identify the PDB proteins (templates) having similar global structures. The global structure similarity between the target and template structures was measured by TM-align [56]. The PDB protein searches identified a PDB entry, 4hqp (α7 nicotinic receptor chimera in complex with α-bungarotoxin, Figure A in S1 Fig) containing templates for both targets in the single PDB entry. A α4:α4/lynx1 complex model (Figure B in S1 Fig) was built by mapping the α4 subunit and lynx1 onto α7 subunits and α-bungarotoxin, respectively.

The α4 subunit structure has a closed-in conformation of loop C. In addition, the structure of lynx1 has more structured loop I, compared to other members of lynx family (e.g., CD59, candoxin, erabutoxin-a) [45]. These structural features result in a number of bad contacts in the initial low-resolution α4:α4/lynx1 complex model. The model was refined through minimizing the bad contacts. We sampled thermally acceptable 100 conformations for α4 loop C and lynx1 loop I using Backrup application in Rosetta [57], generating 100 × 100 combinations of α4:α4/lynx1 complexes with different loop conformations. For each complex, lynx1 was moved outwards from α4:α4 by ≤ 2 Å using an interval of 1 Å along a vector defining the geometric centers of two protein binding surfaces to find a complex structure with the minimum number of bad contacts. The refined α4:α4/lynx1 complex model was subjected to 150-ns molecular dynamics (MD) simulations to obtain a high-resolution complex model. During the simulations, weak positional restraints were applied to the backbone heavy atoms of α4:α4, aiming at maintaining the pentameric orientation of α4:α4. For lynx1, we applied or removed the positional restraints every 10-ns to prevent dissociation of lynx1 from α4:α4. No restraints were applied to loop B, loop C, and β8-β9 loop in α4:α4 and loop I in lynx1, allowing their conformational flexibility, as they might involve protein-protein interactions. After the 150-ns simulations, we removed all the positional restraints to lynx1 and performed additional 100-ns simulations for equilibration. All MD simulations were prepared through CHARMM-GUI [58–59] and performed with explicit water molecules and 150 mM KCl for physiological salt concentration using CHARMM36 force filed [60] and NAMD software [61] at 303.15 K. The final snapshot was energy-minimized and used to describe atomic level interactions between α4:α4 and lynx1 in the complex model.

To generate a β2:β2/lynx1 complex model, we used the same template-based modeling approach based on PDB: 4hqp and simulation protocol as α4:α4/lynx1. In the case of β2:β2/lynx1, however, we did not perform the additional 100-ns equilibration MD simulations. For a structure comparison of α4:α4/lynx1 with β2:β2/lynx1, lynx1 structure in the final α4:α4/lynx1 complex model was copied onto β2:β2 to generate the β2:β2/lynx1 complex model.

### Data presentation and statistical analyses

Hot-plate, locomotion and body temperature data were presented as mean ±S.E.M. Data were analyzed using either the Student T-test or the two-way ANOVA test with a Tukey post-hoc test. All assumptions for the Student’s T-test and two-way ANOVA were considered. A p-value less than 0.05 was considered statistically significant. All statistical tests were performed in IBM SPSS Statistics 24 (IBM Corporation, Armonk, NY, USA). The data and statistical analysis comply with the recommendations on experimental design and analysis in pharmacology.

## Results

**Fig 1:**
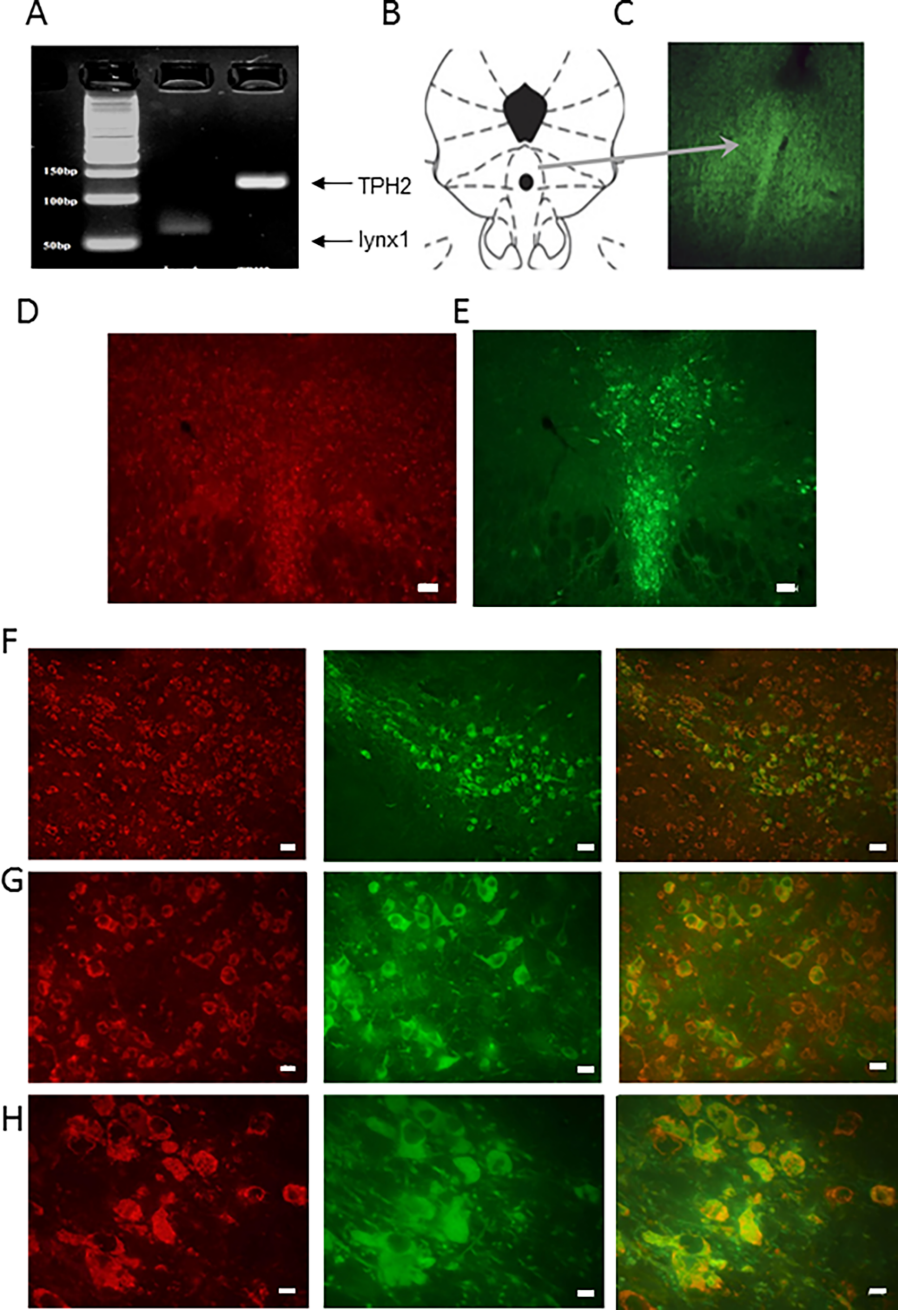
Establishing *lynx1* expression in brain regions associated with nociception. (A) Evidence of *lynx1* mRNA expression in the dorsal raphe nucleus via RT-PCR using *lynx1*-specific primers (expected band size of 62 bp). Expression of TPH2 (expected band size of 147 bp) validates that the isolation is in the correct region of interest. (B) Schematic of the brainstem at the level of the DRN, as a coronal plane of section (C) Expression of lynx1 protein (green) in the dorsal raphe nucleus, using anti-lynx1 pAb immunofluorescence staining [39, 64]and donkey anti-rabbit Cy2 secondary antibody, imaged at 4x magnification. (D and E) Side by side labeling of lynx1 (anti-lynx1 mAb, Alexa red), and TPH2 (anti-TPH2 pAb, Cy2, green), 10x magnification, scale bar = 200 μm. (F) Dual labeling immunofluorescence staining using anti-lynx1 mAb (red) and anti-TPH2 pAb (green), merge (yellow), 20x magnification, scale bar = 100 μm. (G) Dual labeling immunofluorescence staining using anti-lynx1 mAb (red) and anti-TPH2 pAb (green), merge (yellow), 40x magnification, scale bar = 50 μm.

We chose the DRN to begin our investigations because it has been shown to mediate nociceptive signals and express high levels of nAChRs which when activated contribute to antinociception [10]. To determine whether the DRN express *lynx1*, the mRNA expression of *lynx1* and TPH2 in the DRN was analysed using quantitative PCR (relative expression *lynx1*/GAPDH: 0.0024) and Reverse Transcriptase PCR (RT-PCR) (Fig 1A). We chose TPH2 as a marker of the DRN, since it is an enzyme involved in the synthesis of serotonin, and is highly enriched in the DRN [62–63]. Although this establishes the expression of *lynx1* in the DRN, we sought an independent confirmation using another methodology. We also performed immunohistochemical staining using previously published anti-lynx1 antibodies. Immunofluorescence staining using a polyclonal antibody specifically shown to label lynx1 protein [39, 48, 50], showed expression of *lynx1* in ventral regions of the DRN as compared to dorsal regions of the DRN (Fig 1B and 1C) or outside the DRN (Figure C in S1 Fig). Side by side comparative staining of anti-*lynx1* monoclonal [44], and anti-TPH2 polyclonal antibody (Fig 1D and 1E), showed similar regions of staining of TPH2 and *lynx1* in the region of the DRN. Co-labeling studies with these same antibodies showed areas of dual labeling of *lynx1* and TPH2, as well as *lynx1* staining which were not labeled with TPH2 at 10x (Fig 1F), 20x (Fig 1G), and 40x (Fig 1H) magnification. This indicates that *lynx1* is expressed in both serotonergic and non-serotonergic neurons in the DRN.

**Fig 2:**
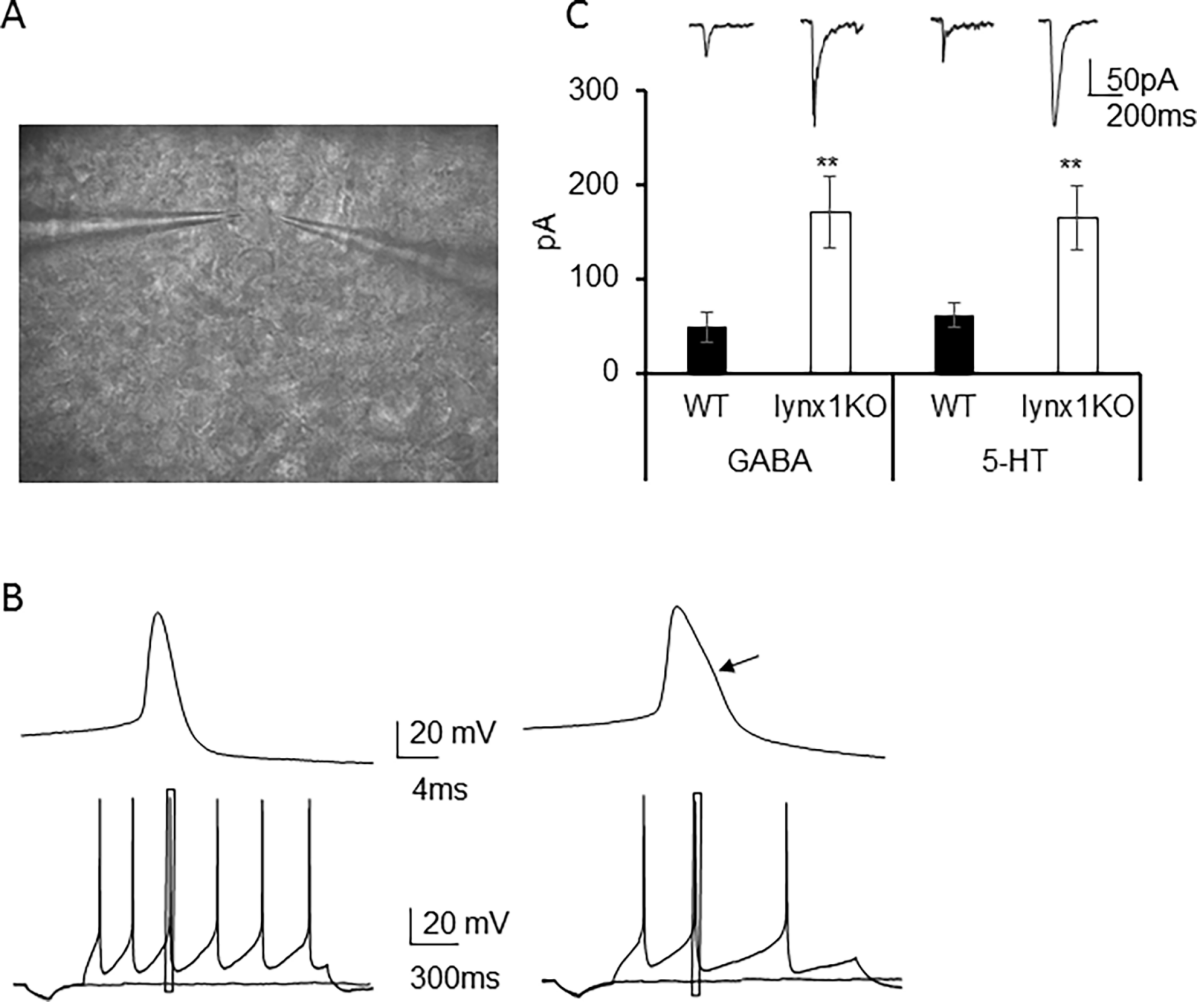
Modulatory effect of *lynx1* on nicotine responses in the dorsal raphe nucleus. (A) Photomicrograph of a live brain slice containing the dorsal raphe nucleus (B) Representative trace of an action potential from a GABAergic-like (without AP shoulder, left) and serotonergic-like neuron (with AP shoulder, right) recorded in current-clamp mode. Left upper panel is a single action potential at a faster time scale and lower panel is a spike train at high frequency of the GABAergic neuron. Right upper panel is a single AP at a faster time scale, and lower panel is a spike train at low frequency of the serotonergic neuron. Arrow points to the AP shoulder which is a hallmark of serotonergic neurons. (C) Representative original traces and average nicotine-evoked current amplitude of neuronal cell bodies recorded in voltage-clamp mode held at -70mV. Nicotine induced stronger responses both in GABAergic and serotonergic neurons of *Lynx1* KO (white) neurons than wild-type (black) in dorsal raphe nucleus (GABAergic neurons: wt 47.8 ± 16.1 pA, n = 9 (mice N = 3) vs. *lynx1*KO 170 ± 38.2 pA, n = 17 (mice N = 6); p = 0.008, student *t* test. Serotonergic neurons: wt 61.0 ± 13.2 pA, n = 12 (mice N = 4) vs. *lynx1*KO 164 ± 34.6 pA, n = 19 (mice N = 6); p = 0.01, student *t* test).

We next were interested to understand if *lynx1* modulates nAChRs in the DRN, using *lynx1*KO slices to assess nicotine-evoked response properties (Fig 2A). There are two major cell types in the DRN, serotonergic and GABAergic, which can be discriminated by the action potential shape [65], with the serotonergic neuron having a shoulder in the falling phase of the action potential. We obtained whole-cell measurements from these two neuronal cell types in the DRN, the neurons with an AP shoulder being serotonergic-like neurons (serotonergic) and those without an AP shoulder being GABAergic-like (GABAergic) neurons, using these parameters to discriminate them from one another (Fig 2B) [65]. We measured acute responses to fast application of pressure-ejected nicotine (200 μM) in neurons of brain slices in the DRN from wt and *lynx1*KO. We found that there were differences in the peak response between wt and *lynx1*KO neurons (Fig 2C), with higher peak nicotine response in neurons from *lynx1*KO vs wt slices (GABAergic neurons: wt 47.8 ± 16.1 pA, n = 9 (mice N = 3) vs. *lynx1*KO 170 ± 38.2 pA, n = 17 (mice N = 6); p = 0.008, student *t* test. Serotonergic neurons: wt 61.0 ± 13.2 pA, n = 12 (mice N = 4) vs. *lynx1*KO 164 ± 34.6 pA, n = 19 (mice N = 6); p = 0.01, student *t* test), in response to fast application of nicotine between the two genotypes and in both cell types (Fig 2C). These data demonstrate a modulatory action of *lynx1* on nicotinic receptors in one of the brain regions involved in nociceptive signaling.

**Fig 3:**
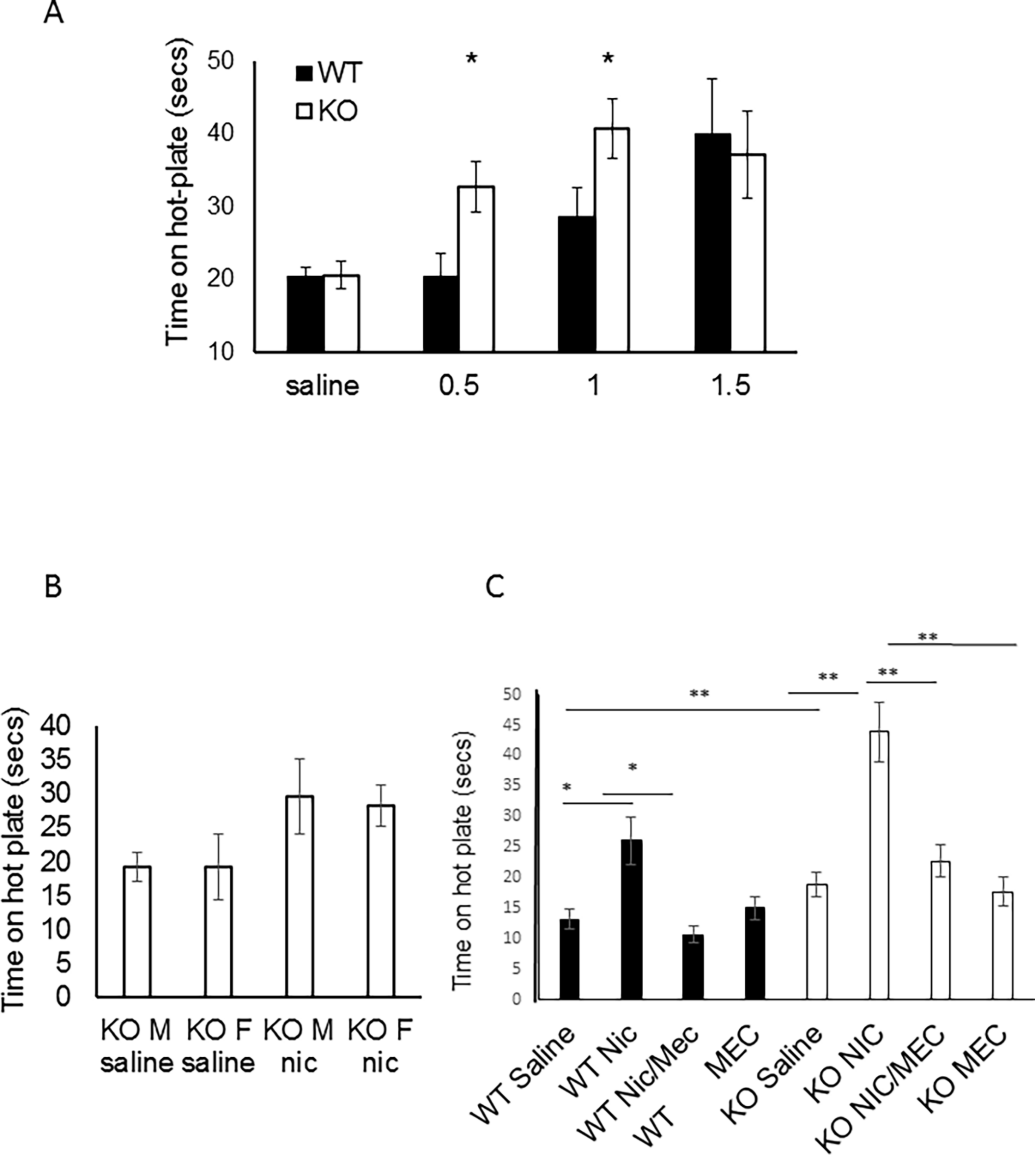
The effect of nicotine on antinociception assessed on a hot-plate assay. (A) Antinociceptive responses in wt and *lynx1*KO mice after I.P. injections of saline (n = 8 wt, 8 KO. p = 0.899, two-way ANOVA, cohen’s D 0.13) or nicotine concentrations of 0.5 mg·kg^-1^ (n = 8 wt, 18 KO. p = 0.122, two-way ANOVA, cohen’s D 1.36), 1.0mg·kg^-1^ (n = 8 wt, 14 KO. p = 0.032, two-way ANOVA, cohen’s D 1.09) and 1.5mg·kg^-1^ (8 wt, 8 KO. p = 0.657, two-way ANOVA, cohen’s D 0.13) using the hot-plate assay. ED_50_ was 1.05 mg·kg^-1^ for wt and 0.44 mg·kg^-1^ for the lynx1KO group.

To address this possibility, we sought to understand the effect of *lynx1* in our genetic mice using a behavioral test of acute thermal nociception. *Lynx1*KO mice have been shown to be more sensitive to the effects of nicotine in electrophysiological, motor learning assays [48] and visual evoked responses in the visual cortex [50] and therefore we predicted that the *lynx1*KO mice would be augmented in either baseline nociceptive responses and/or sensitivity to the antinociceptive effects of nicotine. We conducted thermal assays in wt and *lynx1*KO mice using a hot-plate paradigm. The sensitivity to the heat from the hot-plate was assessed by measuring the response latency to react (secs) under saline and nicotine conditions. No differences in baseline (saline) nociception between wt and *lynx1*KO mice were found (mean difference 0.8 sec, p = 0.899, two-way ANOVA. n = 8, 8; wt, *lynx1*KO, respectively. Cohen’s D 0.13). Both genotypes, however, showed a dose-dependent increase in time spent on the hot-plate after nicotine administration (Fig 3A, Figure A in S2 Fig). *Lynx1*KO mice showed more sensitivity to nicotine as compared to wt mice at lower doses (0.5 mg·kg^-1^ nicotine; mean difference 13.4 sec, p = 0.034, two-way ANOVA, n = 8, 9, Cohen’s D 1.37. 1.0 mg·kg^-1^ nicotine; mean difference 12.2 sec, p = 0.034, two-way ANOVA, n = 8, 14, Cohen’s D 1.09). These data suggest that genetic removal of *lynx1* in mice increased the antinociceptive actions of nicotine. We saw no differences due to sex (Fig 3B).

Next, we blocked nAChRs using a non-selective channel blocker of nAChRs, mecamylamine, in the same hot-plate assay. We injected nicotine (1 mg·kg^-1^) 10 minutes after mecamylamine (2.5 mg·kg^-1^) injection. We found that blocking nAChRs with mecamylamine was able to inhibit the augmentation in nicotine-mediated antinociception observed in both wt and *lynx1*KO mice (Fig 3C) to normal levels of responsiveness similar to saline injected controls (nicotine-treated *lynx1*KO vs. nicotine+mec treated *lynx1*KO mice: mean difference 21.1 sec, p = 0.0001, Student’s T-test, nicotine-treated *lynx1*KO vs. mec treated *lynx1*KO mice: mean difference 26.15 sec, p<0.00001, Student’s T-test, nicotine-treated wt vs. nicotine+mec treated wt mice, mean difference 15.4 sec, p = 0.002, saline treated wt vs nicotine-treated wt, mean difference 12.89, p = 0.014, saline-treated KO vs saline-treated KO nicotine, mean difference 25.0 sec, p<0.00001). All mice were examined for tissue damage post experiments, and no sign of tissue damage or discomfort were observed. These data suggest that that the augmented antinociceptive effect of nicotine in the *lynx1*KO mice is mediated through nAChRs, and that binding of *lynx1* to this receptor modulates its antinociceptive properties.

Mice were tested on the hot-plate 15 minutes after injection. Nicotine-mediated antinociception is augmented in *lynx1*KO mice at nicotine concentrations 0.5 mg·kg^-1^ and 1.0mg·kg^-1^ compared to wt mice. Each data point presented as mean ± SEM. *P<0.05 compared to wt controls at corresponding concentrations of nicotine. wt: wild type, KO: *lynx1* knockout. (B) The effect of nicotine on antinociception in male and female *lynx1*KO mice. Antinociceptive responses in male and female *lynx1*KO mice after I.P. injections of 0.5 mg·kg^-1^ nicotine (n = 8 male, n = 8 female, p = 0.844, student’s t-test) using the hot-plate assay. Mice were tested on the hot-plate 15 minutes after injection. There are no antinociceptive difference between sexes. Each data point presented as mean ± SEM. (C) Antinociceptive responses in wt and *lynx1*KO mice after I.P. injection of the general nAChR blocker, mecamylamine (2.5 mg·kg^-1^) and nicotine (1mg·kg^-1^) (nicotine treated *lynx1*KO mice (n = 14) vs. nicotine+mecamylamine treated *lynx1*KO mice (n = 13): p = 0.003, Student’s T-test and nicotine treated wt mice (n = 8) vs. nicotine+mecamylamine treated wt mice (n = 16): p<0.001, Student’s T-test) using the hot-plate assay. Injections of mecamylamine block the antinociceptive effect of nicotine in both genotypes. Data indicates that *lynx1* operates through nAChRs. Data presented as mean ± SEM time. *P<0.05 nicotine injected animals compared to nicotine+mecamylamine injected animals. wt: wild type (C57bl6, *lynx1* +/+ and β2-GFP), KO: *lynx1* knockout. We performed statistical analysis between sex, and no significant differences were seen between female and male mice (Fig 3B).

**Fig 4:**
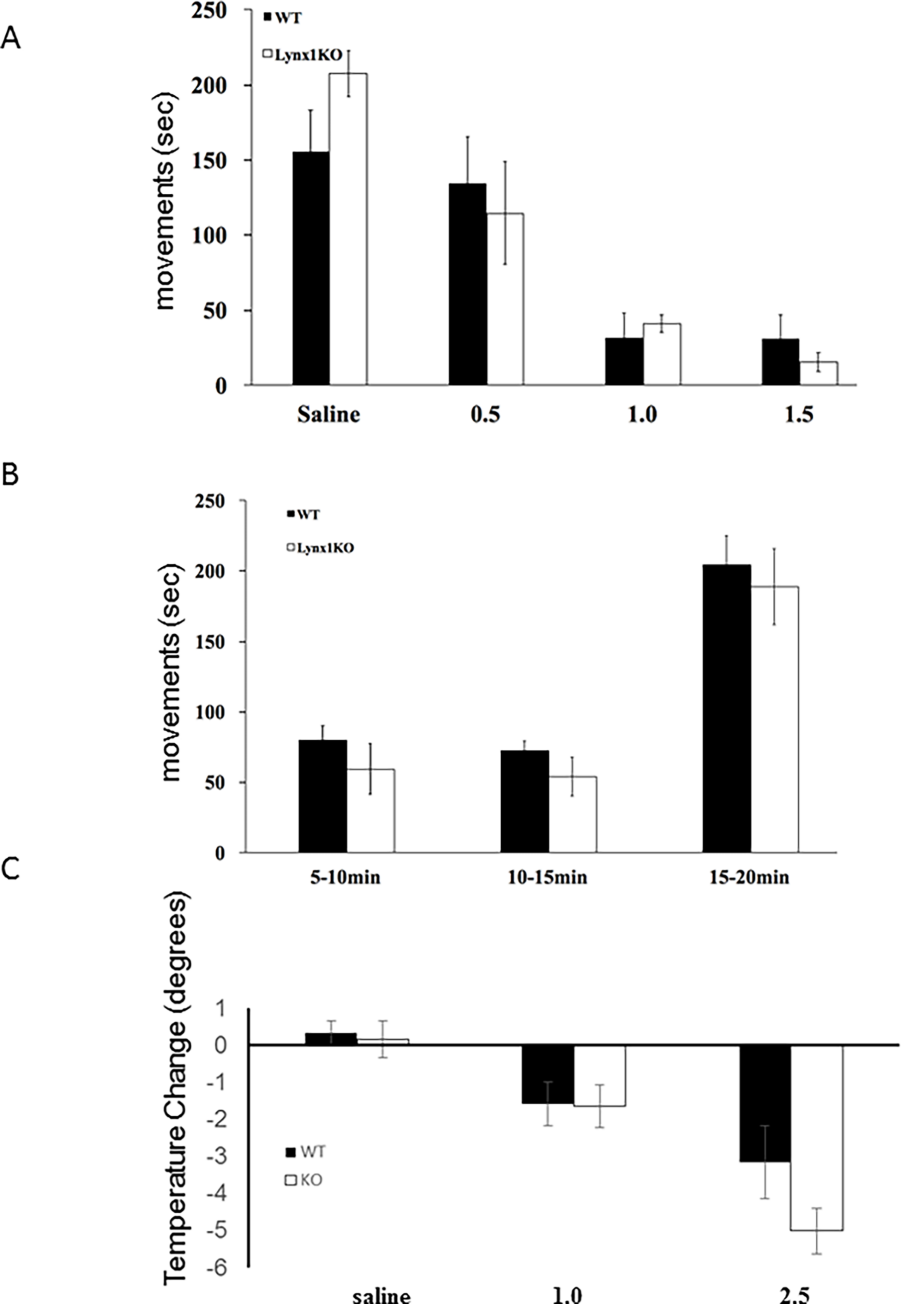
*lynx1* does not influence nicotine-mediated locomotor performance or body temperature. (A) Effect of nicotine on locomotion in wt and *lynx1*KO mice after I.P. injections of nicotine concentrations 0.5 mg·kg^-1^ (n = 7 wt, 8 KO), 1.0mg·kg^-1^ (n = 6 wt, 6 KO), 1.5mg·kg^-1^ (6 wt, 6 KO). Locomotion were examined by scoring leg movements (seconds) in the time period 15–20 minutes post injection. Injection of nicotine induce the same amount of hypolocomotion in both genotypes. Each data point presented as mean ± SEM. wt: wild type, KO: *lynx1* knockout. (B)The locomotor performance after nicotine injection (0.5 mg·kg^-1^) was binned into 5 minute time windows and showed no significant effect of genotype at any time window. (C) Effect of nicotine on body temperature in wt and *lynx1*KO mice after I.P. injections of either saline (n = 11 wt, 11 KO), or nicotine concentrations 1.0 mg·kg^-1^ (n = 9 wt, n = 11 KO) and 2.5 mg·kg^-1^ (n = 7 wt, 7 KO). Each bar presented as mean ± SEM.

The effects of nicotine on locomotion have been well-documented [66] and could bias the antinociception data presented. We therefore sought to determine if changes in response time on the hot-plate could be due to nicotine mediated changes in voluntary movements. To carry this out, we tested locomotor activity in nicotine injected animals at the same doses used in the hot-plate experiments. There was a dose-dependent decrease in movement in response to nicotine exposure (nicotine-treated wt: 0.5 mg·kg^-1^ 114.55±34.37 sec n = 7, 1.0 mg·kg^-1^ 31.46±16.39 sec n = 6, 1.5 mg·kg^-1^ 31.09±15.43 sec n = 6 and nicotine treated *lynx1*KO: 0.5 mg·kg^-1^ 134.54±21.05 sec n = 8, 1.0 mg·kg^-1^ 41.13±5.81 sec n = 8, 1.5 mg·kg^-1^ 15.46±6.59 sec n = 8 in movement from t15-t20 post drug administration), but no differences between wt and *lynx1*KO mice were observed (Fig 4A). In particular, the 0.5 and 1.0 mg·kg^-1^ dose of nicotine (nicotine-treated wt vs. nicotine-treated *lynx1*KO mice: 0.5 mg·kg^-1^ nicotine; mean difference in movement from t15-t20 post drug administration: 19.9 sec, p = 0.56, two-way ANOVA, 1.0 mg·kg^-1^ nicotine; mean difference in movement from t15-t20 post drug administration mean difference: 9.7 sec, p = 0.778, two-way ANOVA), which improved antinociception in *lynx1*KO mice in the hot-plate experiment (Fig 4A). In addition, in a small subgroup, we analyzed locomotor activity in the time period 5–10, and 15–20 minutes post injection, for saline and 0.5 mg·kg^-1^ nicotine, to confirm that no difference in locomotor activity between genotypes was observed at any relevant time period (Fig 4B, Figure C in S2 Fig). This is consistent with previous reports in other locomotor assays, such as the rotarod motor coordination test [48]. We also investigated nicotine-mediated hypothermia between wt and *lynx1*KO mice and observed a dose-dependent change in temperature (p = 0.00027), but no effects between genotype on nicotine-induced drop in temperature at any of the doses tested (before injection–after injection) (nicotine-treated wt: 0.0 mg·kg^-1^ (saline), 0.32±0.33°, n = 11, 1.0 mg·kg^-1^, -1.60±0.60°, n = 9, 2.5 mg·kg^-1^, -3.2±0.98°, n = 7, and nicotine-treated *lynx1*KO: 0.0 mg·kg^-1^, 0.15±0.5°, n = 11 p = 0.79, t-test, 1.0 mg·kg^-1^, -1.66±0.60°,n = 11 p = 0.47 mg·kg^-1^, 2.5 mg·kg^-1^, -5.0±0.61°, n = 7, p = 0.135, t-test). (Fig 4C). Therefore, the increased latency on the hot-plate in *lynx1*KO mice is likely due to a specific effect of nicotine-induced thermal antinociception rather than nicotine metabolism, or a nonspecific effect of nicotine on systems independent of the nociception pathway.

**Fig 5:**
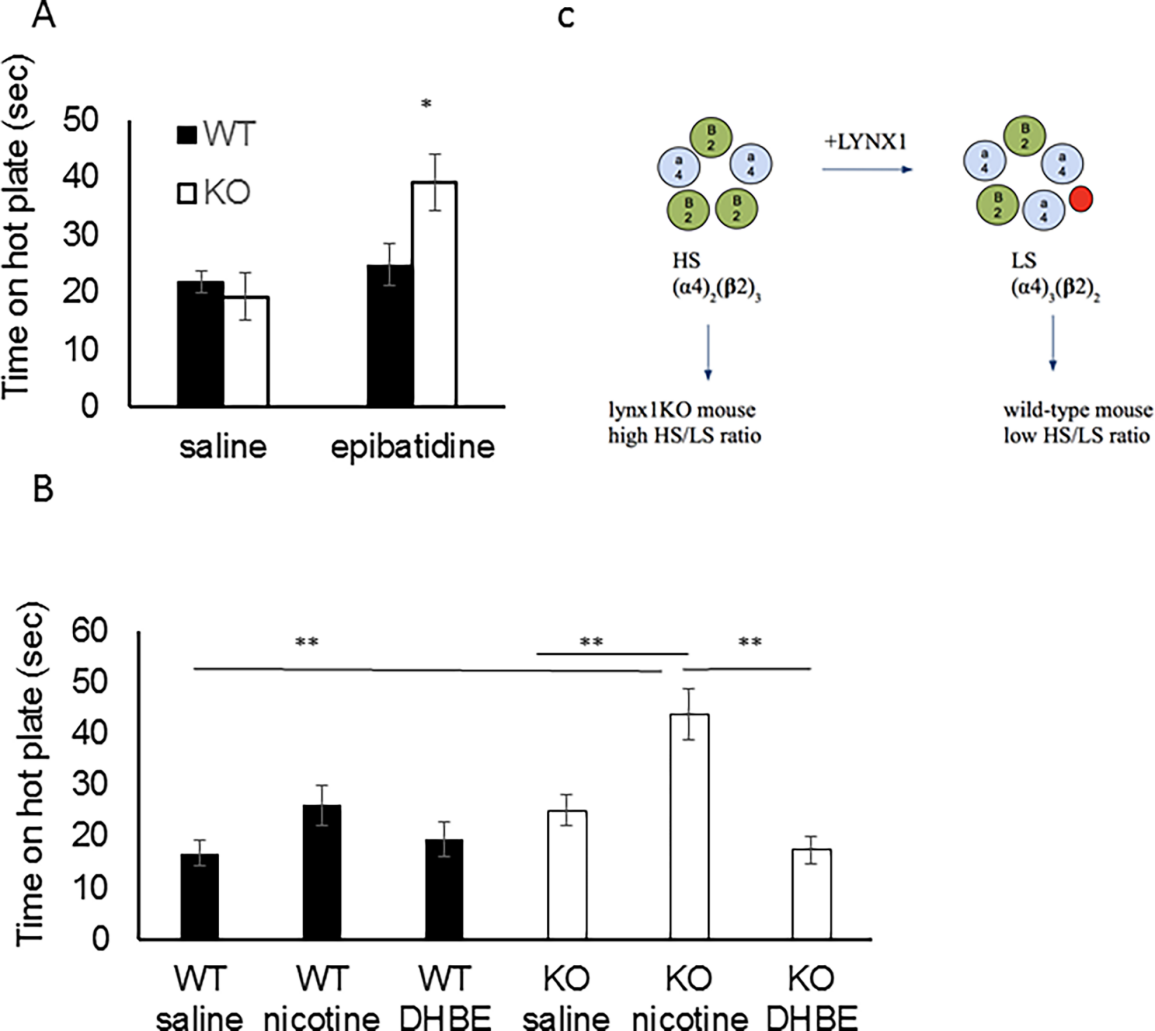
Mediation of *lynx1* through α4β2 nAChRs. (A) Antinociceptive responses in wt and *lynx1*KO mice after I.P. injection of the non-selective α4β2* nAChR agonist, epibatidine (5 μg·kg^-1^) (n = 24 wt, 21 KO, p = 0.029, Student’s T-test). Mice were tested on the hot-plate 15 minutes after injection. Epibatidine-mediated antinociception is augmented in *lynx1*KO mice compared to wt mice. Data presented as mean ± SEM time. *P<0.05 compared to wt controls. wt: wild type, KO: *lynx1* knockout. (B) Antinociceptive responses in wt and *lynx1*KO mice after I.P. injection of the α4β2 nAChRs inhibitor dihydro-β-erythroidine hydrobromide (DHβE) (3.0 mg·kg^-1^) and nicotine (0.5 mg·kg^-1^) (nicotine treated *lynx1*KO mice (n = 8) vs. nicotine+DHβE treated *lynx1*KO mice (n = 6) using the hot-plate assay. Mice were injected with DHβE 25 minutes and nicotine 15 minutes prior to hot-plate testing. Injections of DHβE blocks the antinociceptive effect of nicotine in *lynx1*KO mice. Data indicates that lynx1 operates through the α4β2 nAChR to modulate antinociception. Data presented as mean ± SEM time. wt: wild type, KO: *lynx1* knockout. (C) Schematic of lynx1 binding to the LS stoichiometry of α4β2 nAChRs preferentially over the HS stoichiometry. α4β2 nAChR pentamers shown in the high sensitivity (HS) and low sensitivity (LS) stoichiometry, made up of (α4)_2_(β2)_3_ vs. (α4)_2_(β2)_3_ nAChRs respectively. In our model, lynx1 preferentially binds and stabilizes the LS stoichiometry.

Since lynx1 has been shown to bind to the α4β2 nAChR subtype [44, 47], it is possible that the modulating effect of *lynx1* on nociception could be mediated through the α4β2 nAChR subtype. Epibatidine is a non-selective α4β2* nAChR agonist [8]), which has higher affinity for α4β2 over α7 nAChR subtypes [67–68], and preferential affinity at the low sensitivity (LS) (α4)_3_(β2)_2_ stoichiometry of the α4β2 nAChR [69]. We tested the effect of epibatidine and found a significant increase in time on the hot-plate in *lynx1*KO mice compared to wt mice (epibatidine-treated wt vs. epibatidine treated *lynx1*KO mice: mean difference 20.0 sec, p = 0.029, Student’s T-test) (Fig 5A), suggesting that lynx1 operates through α4β2* nAChRs to modulate their sensitivity to agonist. We tested the effect of blocking β2* nAChRs using the selective antagonist dihydro-β-erythroidine hydrobromide (DHβE) [70]. We injected mice with nicotine (1.0 mg·kg^-1^) 10 minutes after DHβE (3.0mg·kg^-1^) and tested the mice on the hot-plate. We found that DHβE was able to significantly block nicotine’s antinociceptive effect in *lynx1*KO mice, which suggests that that this effect is mediated through α4β2 nAChRs (ANOVA, p < 0.001, F = 7.51_(5,83)_, Tukey post-hoc test, nicotine-treated *lynx1*KO vs. nicotine+DHβE treated *lynx1*KO mice, mean difference 26.56 secs, p = 0.001, nicotine-treated wt vs nicotine-treated KO, mean difference 27.13 secs, p<0.001) (Fig 5B).

To confirm the interaction of lynx1 with α4β2 nAChRs, we performed co-immunoprecipitation experiments using β2-GFP mice [71] to pull out native nAChR complexes from the brain. We used anti-GFP antibodies to pull-down nAChRs and associated protein, and lynx1 as an associated part of the complex was probed using anti-lynx1 antibodies and Western blot analyses. We could detect lynx1 present at enriched levels in the pull-down lane, whereas the flow-through lane was blank (Figure D in S1 Fig), suggesting that lynx1 forms a stable complex with β2*-containing nAChRs. This in support of recent reports of lynx1 in aiding the assembly and stoichiometry of α4β2 receptors [47]. In that report, there was an apparent shifting to the low sensitivity stoichiometry of (α4)_3_(β2)_2_ by a preferential binding affinity of lynx1 to the α4:α4 interface (Fig 5C). In order to gain insight into this idea, we employed computational modeling to address stoichiometry-specific interactions.

**Fig 6:**
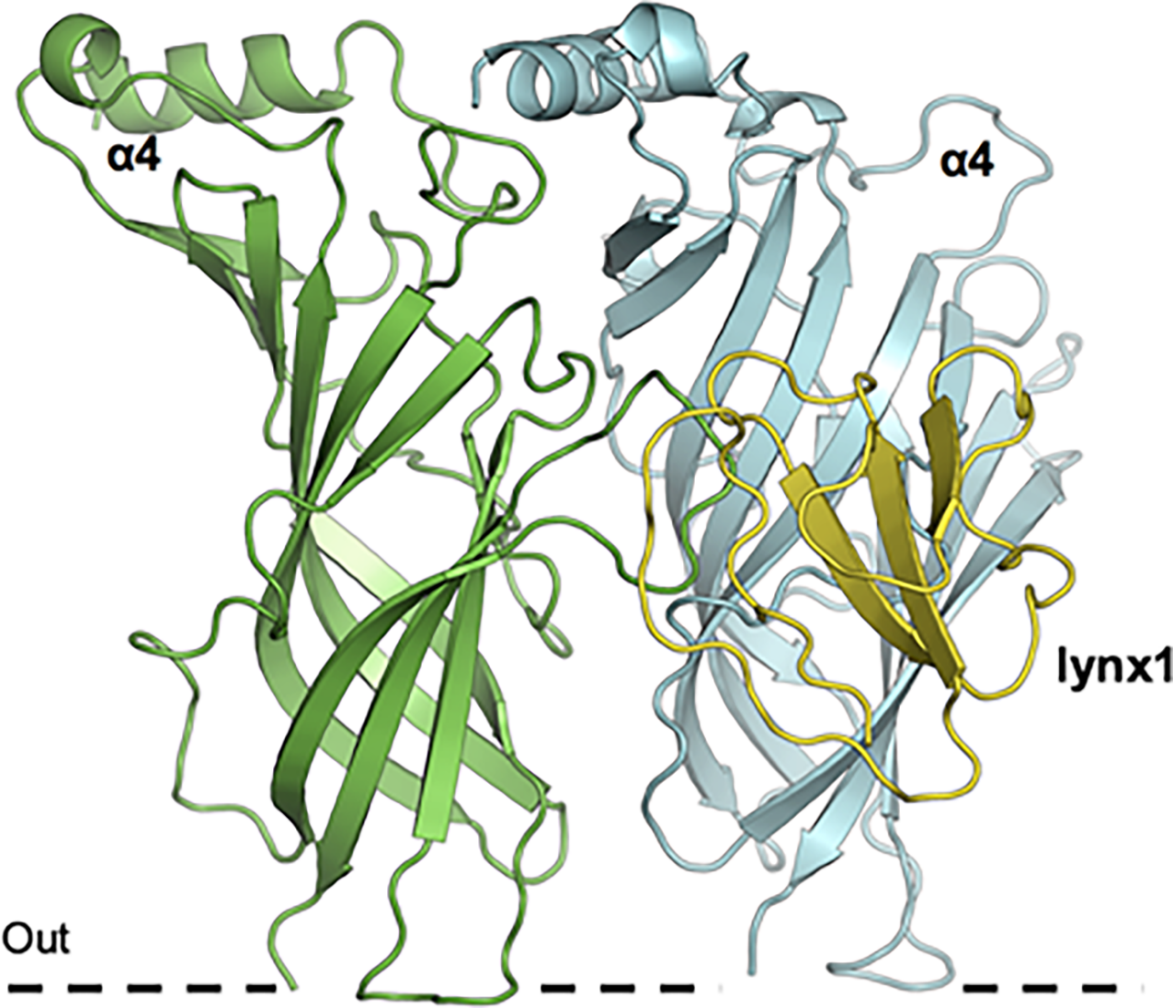
Overall architecture of lynx1 at α4:α4 nAChR interface. Molecular dynamic simulations of lynx1 binding to α4 nAChR subunits. The cell membrane is represented as a dashed line.

We performed simulations of lynx1 binding to the interfaces of the unique interfaces of the α4β2 receptor stoichiometries: α4:α4 vs. β2:β2. The overall architecture of lynx1 at the α4:α4 interface is shown in Fig 6.

**Fig 7:**
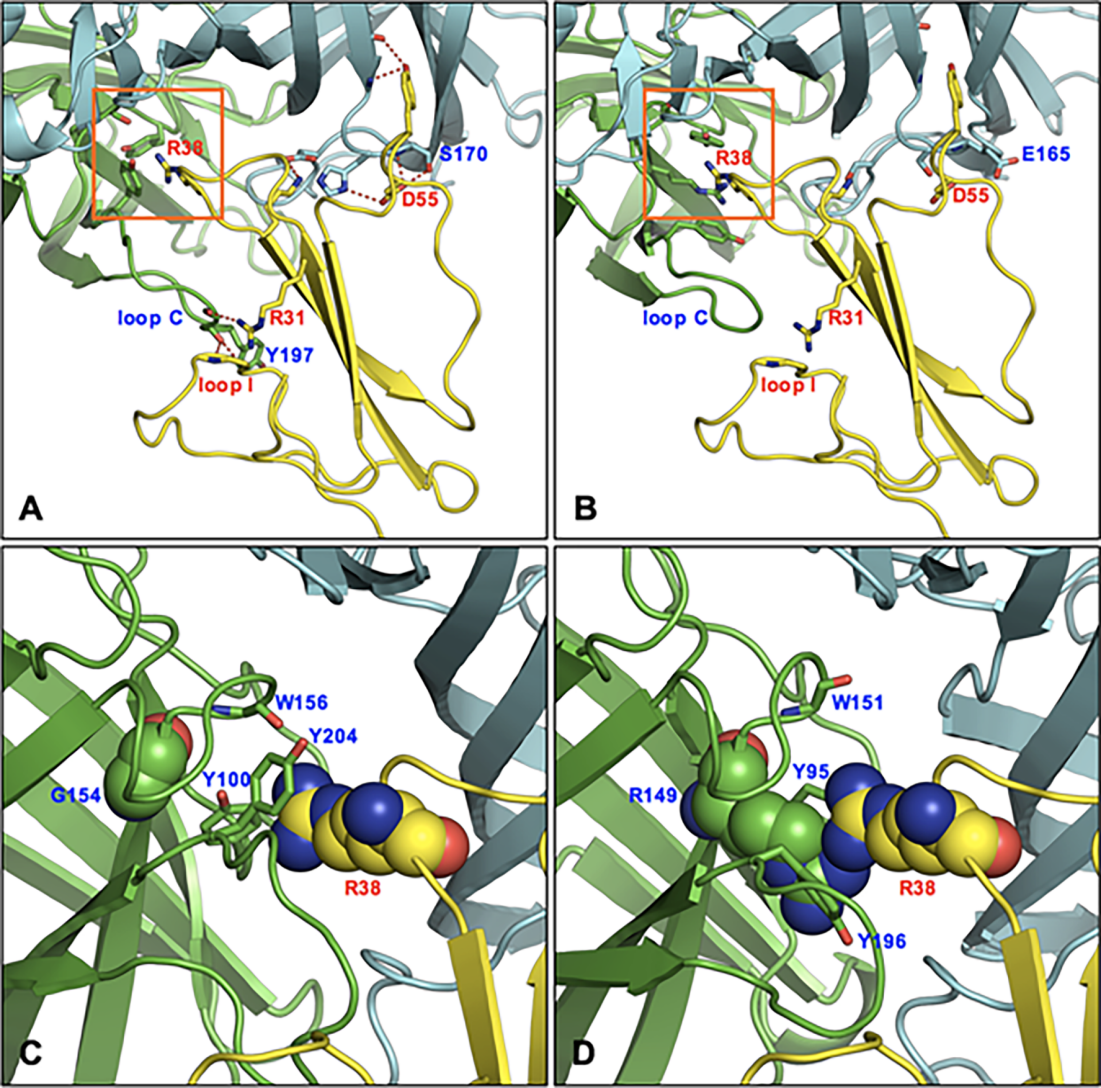
Structural comparison between α4:α4/lynx1 and β2:β2/lynx1 complex model. (A) α4:α4/lynx1 complex model. (B) β2:β2/lynx1 complex model (C) Structural details of boxed in A. (D) Structural details of boxed in B.

From comparisons between α4:α4 and β2:β2 nAChRs structures in complex with lynx1, we found that more favorable interactions exist between the α4:α4 interface and lynx1 (Fig 7). In the α4:α4/lynx1 complex model (Fig 7A and 7C), lynx1 D55 forms three hydrogen bonds with two residues (S170 and H169) in the α4 subunit, while two hydrogen bonds in α4 subunit are due to substitution of E165 in the β2 subunit for S170 in the α4 subunit. Loop C of the α4 nAChR subunit closely contacts with loop I of lynx1, forming hydrogen bonds and hydrophobic interactions (α4 Y197 with lynx1 V6 and M18). Lynx1 R31 also interacts with nAChRs loop C through a hydrogen bond. On the contrary, loop C of the β2 subunit (Fig 7B and 7D) is shorter, making it difficult to generate favorable interactions with lynx1 loop I. In particular, G154 in the α4 subunit is replaced by R149 at the equivalent position in the β2 subunit, implying that the presence of this positive charged residue could prevent lynx1 R38 from approaching and interacting with Y95, W151, and Y196. In the α4:α4/lynx1 complex model, R38 appears to form hydrogen bonds with Y100 and W156, and cation-π interaction with Y204 [72]. Therefore, our modeling and simulations support the experimental results of preferential binding affinity of lynx1 to the α4:α4 interface, as compared to the β:β interface.

More favorable interactions exist between the α4:α4 interface and lynx1; lynx1 D55 forms three hydrogen bonds with two residues (S170 and H169) in the α4 subunit, while two hydrogen bonds in the α4 subunit are due to substitutions of E165 in the β2 subunit for S170 in the α4 subunit. Loop C of the α4 subunit closely contacts with loop I of lynx1, forming hydrogen bonds and hydrophobic interactions (α4 Y197 with lynx1 V6 and M18). Lynx1 R31 also interacts with loop C through a hydrogen bond. On the contrary, loop C of the β2 subunit is shorter, making it difficult to generate favorable interactions with lynx1 loop I.

The lynx1 protein and receptor is colored yellow and green/cyan, respectively. Labels for α4:β2 and lynx1 are colored blue and red, respectively. Potential hydrogen binding are represented as dashed red lines.

## Discussion

In this study we discovered that *lynx1* is expressed in at least one region of the nociception pathway in the CNS, and that mice without the *lynx1* gene demonstrated more nicotine-mediated antinociception. Peak nicotine-evoked responses were larger in *lynx1*KO neurons in the DRN. Furthermore, the data suggest that a possible mechanism of antinociception in the *lynx1*KO is mediated through the α4β2 nAChR subtype. The data are congruent with the hypothesis that nAChRs of *lynx1*KO mice are more sensitive to the effects of nicotine than wtanimals, resulting in an improvement in cholinergic-mediated behavior; in this case antinociception.

Serotonergic neurons in the DRN are well established components in nociception pathways and our data demonstrate the presence of *lynx1* in DRN neurons (Fig 1) [28, 73–75]. Hypersensitive nAChR receptors in both serotonergic and GABAergic neurons caused by *lynx1* removal could shift the excitatory-inhibitory balance towards antinociception. This does not exclude the role of other brain regions, however, as *lynx1* is widely expressed in the CNS [18]. Also, our data do not exclude a role of peripheral sites expressing nAChRs in the nociceptive behavior. *Lynx1* has not been detected in high levels in the periphery, but the effects of *lynx1* outside the CNS awaits further testing. Although our data demonstrate that *lynx1*KO mice are more sensitive to the effects of nicotine in the hot-plate assay than wt mice, we do not exclude effects of endogenous acetylcholine acting in antinociception in lynx1KO mice. Since we do not see an effect of a non-nicotinic drug (e.g ibuprofen), (Figure C in S2 Fig) the role of endogenous acetylcholine would likely be subtle, indicating another example of elevated cholinergic tone in these mice [18,50].

Although the precise mechanism by which lynx1 influences antinociception is as yet unknown, lynx1 has been shown to have global and multiple effects on nAChR function [44, 47–49], so there are candidate mechanisms. The role of specific nAChRs in the DRN and nociception has been demonstrated [36]. Our data are in line with reports about the involvement of α4β2 nAChRs in antinociception [10, 11, 76–77], but does not exclude the involvement of other nAChR subtypes [20, 23, 24, 78]. Lynx1 has also been shown to act on α5α3β4 nAChRs [49], but an involvement at other α4β2* nAChRs (e.g., α5α4β2, α6α4β2, etc.) has yet to be tested. In this model, we have not included the role of α7 nAChRs, which has been shown to mediate nociceptive signaling and to bind to lynx1 proteins [22, 44]. Thus, further research into the specific subtypes involved is needed.

The biophysical mechanism of lynx1 action has been hypothesized to be due to interactions at the interface of subunits of the nAChR pentamer. Lynx1 has also been shown to have global allosteric actions on nAChR function. This includes, not only orthosteric effects on agonist sensitivity, but also includes effects on desensitization kinetics, recovery from desensitization [44, 48] and nAChR receptor closed times [49]. The antinociceptive actions of lynx1 removal could be explained by a lynx1-directed shift in the stoichiometry of the α4β2 nAChRs from a HS to a LS [47] (Fig 4B). The HS nAChR, adopting the (α4)_2_(β2)_3_ stoichiometry, exhibits higher sensitivity and slower desensitization, whereas the LS nAChR, adopting the (α4)_3_(β2)_2_ stoichiometry, exhibits lower sensitivity to agonist, faster desensitization and higher Ca^2+^ permeability [69,79] In the lynx1KO mice, we expect a higher relative expression of the HS stoichiometry, which is supported by our data which show an enhanced sensitivity to nicotine in lynx1KO mice as compared to wt mice. The computer simulation data further support the HS stoichiometry by (Figs 6 and 7) suggesting a preferential binding of lynx1 to the α4:α4 interface of the α4β2 nAChRs. We also saw that the β2*-selective nAChR antagonist DHβE was able to block nicotine’s antinociceptive effect in lynx1KO mice, which further suggests that lynx1 is able to modulate the α4β2 nAChR subtype, demonstrating the β2* nAChRs involvement in antinociception but not locomotion or thermal regulation [80] (Fig 4).

Untreated acute pain can influence the development of chronic pain and can have deleterious effects on quality of life and account for billions in hospital costs [81, 82]. Thus, management of acute pain is essential for the patient and the public health in both the short and long term [83–84]. Our data addresses acute pain more so than chronic or neuropathic pain, but the relationship of lynx1 to plasticity suggests that other types of pain (neuropathic, peripheral, chronic, etc.) might be altered in our genetic model. A recent paper reported that epibatidine’s antinociceptive efficacy declined over time in a mouse model of chronic pain and that no changes in the number and affinity of α4β2 nAChRs occurred [85], suggesting the possibility that lynx1 may modulate behavioral tolerance to nicotine. However, this is speculative and further investigation is needed.

Summary: nAChRs have been implicated in nociceptive processing and has been explored as a therapeutic avenue for the alleviation of nociception. The modulator lynx1 can regulate nicotinic activity through its ability to bind and regulate nAChRs. We found a nicotine-mediated antinociception involving α4β2 nAChRs, which is augmented in lynx1KO mice.

## Supporting information

S1 Fig Lynx1/nAChR interaction. A. Overall structures of α7/AChBP chimaera (PDB entry: 4hqp). B. Low-resolution α4:α4/lynx1 model. C. Periaqueductal grey immunostaining using anti-lynx1 monoclonal antibody (anti-lynx1 mAb, Alexa red), 10x magnification, scale bar = 200 μm. D. Detection of lynx1/β2 interaction by Western blot analysis after GFP co-immunoprecipitation in β2-GFP mice. Lane 1 is the Co-IP sample. Lane 2 is the Co-IP input. Lane 3 is a wildtype total brain homogenate (TH). Lane 4 control is an untreated total protein homogenate from a wildtype hippocampus.
(TIF)

S2 Fig The effect of nicotine and ibuprofen on antinociception and saline on locomotion. A. Antinociceptive responses in wt and *lynx1*KO mice after I.P. injections of nicotine at concentrations of 0.25 mg·kg^-1^ (n = 8 wt, 8 KO, p = 0.656 two-way ANOVA, cohen’s D 0.40), 0.5 mg·kg^-1^ (n = 8 wt, 18 KO, p = 0.122, two-way ANOVA, cohen’s D 1.36), 1.0mg·kg^-1^ (n = 8 wt, 14 KO, p = 0.032, two-way ANOVA, cohen’s D 1.09) and 1.5mg·kg^-1^ (8 wt, 8 KO. p = 0.657, two-way ANOVA, cohen’s D = 0.13) using the hot-plate assay plotted in a semi-log format. Mice were tested on the hot-plate 15 minutes after injection. Nicotine-mediated antinociception is augmented in *lynx1*KO mice at nicotine concentrations 0.5 mg·kg^-1^ and 1.0mg·kg^-1^ compared to wt mice. Each data point presented as mean ± SEM. *P<0.05 compared to wt controls at corresponding concentrations of nicotine. wt: wild type, KO: *lynx1* knockout.
B. Effect of saline on locomotion in wt and *lynx1*KO mice after I.P. injections of saline (n = 8 wt, n = 8 KO, not significant). Locomotion were examined by scoring leg movements (seconds) in the time period 15–20 minutes post injection. The locomotor performance was binned into 5 minute time windows and showed no significant effect at any time window. Each data point presented as mean ± SEM. wt: wild type, KO: *lynx1* knockout. C. Effect of ibuprofen sodium salt in wt and *lynx1*KO mice after I.P. injection, 20 mg·kg^-1^ (n = 10 wt, n = 12 KO, not significant). Each data point presented as mean ± SEM. wt: wild type, KO: *lynx1* knockout.
(TIF)
