# Supplementary figures and images for: Augmenting the antinociceptive effects of nicotinic acetylcholine receptor activity through lynx1 modulation
Source: PLoS One. 2018 Jul 3;13(7):e0199643. doi: 10.1371/journal.pone.0199643 (PMC6029753; doi:10.1371/journal.pone.0199643)

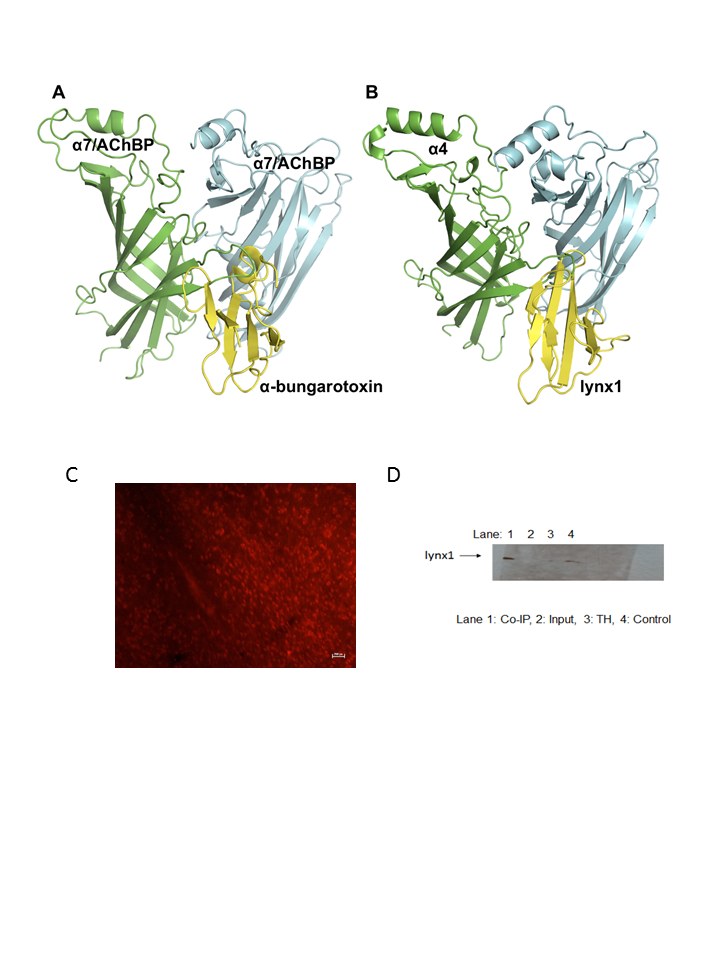

Supplement: S1 Fig — A. Overall structures of α7/AChBP chimaera (PDB entry: 4hqp). B. Low-resolution α4:α4/lynx1 model. C. Periaqueductal grey immunostaining using anti-lynx1 monoclonal antibody (anti-lynx1 mAb, Alexa red), 10x magnification, scale bar = 200 μm. D. Detection of lynx1/β2 interaction by Western blot analysis after GFP co-immunoprecipitation in β2-GFP mice. Lane 1 is the Co-IP sample. Lane 2 is the Co-IP input. Lane 3 is a wildtype total brain homogenate (TH). Lane 4 control is an untreated total protein homogenate from a wildtype hippocampus. (TIF) [file pone.0199643.s001.TIF]

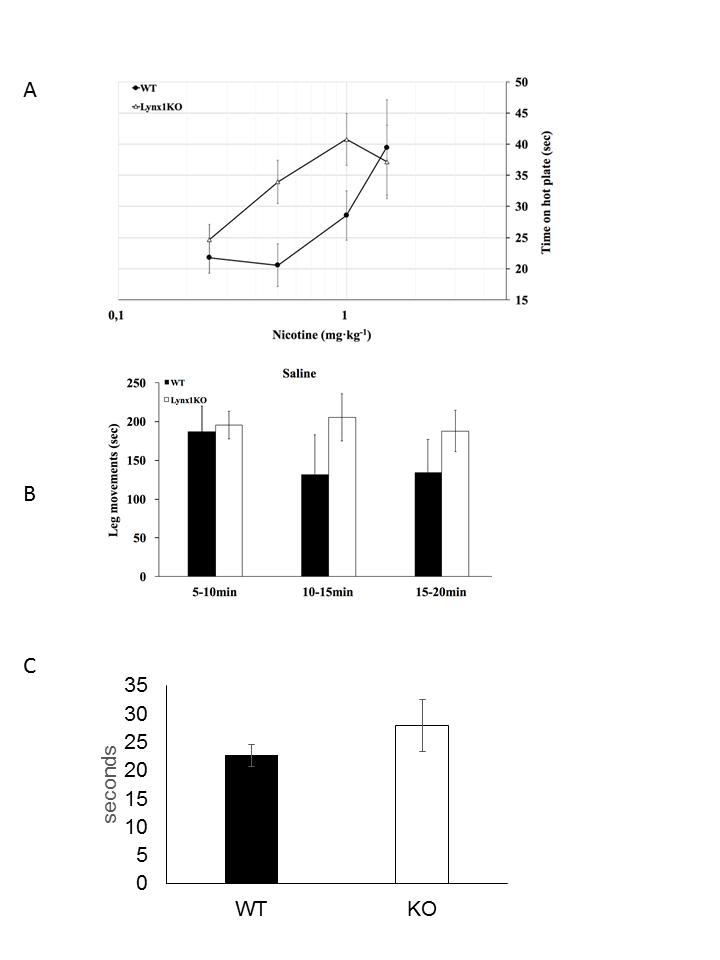

Supplement: S2 Fig — A. Antinociceptive responses in wt and lynx1KO mice after I.P. injections of nicotine at concentrations of 0.25 mg·kg-1 (n = 8 wt, 8 KO, p = 0.656 two-way ANOVA, cohen’s D 0.40), 0.5 mg·kg-1 (n = 8 wt, 18 KO, p = 0.122, two-way ANOVA, cohen’s D 1.36), 1.0mg·kg-1 (n = 8 wt, 14 KO, p = 0.032, two-way ANOVA, cohen’s D 1.09) and 1.5mg·kg-1 (8 wt, 8 KO. p = 0.657, two-way ANOVA, cohen’s D = 0.13) using the hot-plate assay plotted in a semi-log format. Mice were tested on the hot-plate 15 minutes after injection. Nicotine-mediated antinociception is augmented in lynx1KO mice at nicotine concentrations 0.5 mg·kg-1 and 1.0mg·kg-1 compared to wt mice. Each data point presented as mean ± SEM. *P<0.05 compared to wt controls at corresponding concentrations of nicotine. wt: wild type, KO: lynx1 knockout. B. Effect of saline on locomotion in wt and lynx1KO mice after I.P. injections of saline (n = 8 wt, n = 8 KO, not significant). Locomotion were examined by scoring leg movements (seconds) in the time period 15–20 minutes post injection. The locomotor performance was binned into 5 minute time windows and showed no significant effect at any time window. Each data point presented as mean ± SEM. wt: wild type, KO: lynx1 knockout. C. Effect of ibuprofen sodium salt in wt and lynx1KO mice after I.P. injection, 20 mg·kg-1 (n = 10 wt, n = 12 KO, not significant). Each data point presented as mean ± SEM. wt: wild type, KO: lynx1 knockout. (TIF) [file pone.0199643.s002.TIF]
